# Mesoscopic Simulation of the Dynamic Damage and Failure Mechanism of Three-Phase Concrete Under Rigid Projectile Penetration

**DOI:** 10.3390/ma19143078

**Published:** 2026-07-17

**Authors:** Xiaoli Wang, Shutao Li, Yeqing Chen, Shang Ma, Jialin Chen

**Affiliations:** State Key Laboratory of Target Vulnerability Assessment, Institute of Defense Engineering, Academy of Military Sciences (AMS), PLA, Beijing 100036, China; wangxiaoli18@163.com (X.W.); yeqing_chen2020@163.com (Y.C.); sma_1002@hrbeu.edu.cn (S.M.); cjl0321@yeah.net (J.C.)

**Keywords:** concrete, mesoscopic model, rigid elasticity, penetration, dynamic damage

## Abstract

**Highlights:**

- The concrete damaged plasticity (CDP) model considering the strain-rate effect has been improved.
- A self-automatically generated three-phase microscale penetration model for concrete has been established.
- The regulatory mechanism of the microstructure on penetration damage and ballistic behavior has been revealed.

**Abstract:**

This study aims to clarify the mesoscopic damage evolution mechanisms of concrete subjected to rigid projectile penetration and provide support for the optimal design of high-performance protective structures. Based on the ABAQUS/Explicit finite element framework, a three-phase mesoscopic numerical model of concrete considering aggregate, mortar matrix, and interfacial transition zone (ITZ) is constructed. By combining the random convex polygon algorithm with the background mesh mapping technique, the intrinsic geometric features of stochastic materials such as crushed stone and pebble are accurately characterized. The effects of aggregate geometric characteristics, volume fraction, and projectile motion/geometry parameters (velocity, length–diameter ratio, curvature radius of the warhead CRH) on the damage evolution of the target, penetration depth, and velocity attenuation law are systematically investigated. The results reveal that increased aggregate angularity substantially enlarges both tensile and compressive damage zones and promotes crack bifurcation, which collectively enhances kinetic energy dissipation, reduces penetration depth, and accelerates projectile deceleration. Increasing the aggregate volume fraction can significantly enhance the anti-penetration resistance of the target. A high proportion of aggregate grains effectively enhances the structural toughness by blocking the crack propagation path. Penetration velocity, length–diameter ratio, and CRH are the core elements determining the penetration efficiency, and the increase in their values will lead to a significant increase in penetration depth and induce a change in the damage mode from local failure to large-scale cracking. The mesoscopic model and related conclusions established in this study can provide a theoretical foundation and numerical benchmark for the impact resistance design, optimization, and damage assessment of high-strength concrete protective structures.

## 1. Introduction

As a typical multiphase composite material, concrete is widely utilized in critical infrastructure facilities such as national defense protection structures, transportation engineering, and water conservancy hubs. Its penetration resistance directly determines the safety and durability of structures [1]. Penetration is defined as the process where a projectile moving at a certain velocity penetrates into or perforates a target object [2]. Under extreme working conditions, including military conflicts, terrorist attacks, and accidental impacts, concrete structures are frequently subjected to projectile penetration, which triggers cumulative structural damage, a sharp decline in load-bearing capacity, and eventually leads to overall structural failure [3]. Therefore, in-depth investigation of the mesoscopic mechanism of projectile penetration into concrete and clarification of the influence law of various factors on the penetration process are of great theoretical value and engineering significance for optimizing the design of protective concrete structures and improving their penetration resistance.

Since the 20th century, extensive research efforts have focused on experimental investigations of projectiles with different geometric features at impact velocities ranging from 200 m/s to 1000 m/s [4,5]. These early experimental studies have accumulated valuable data regarding DOP, target penetration, cratering effect, and the ricochet phenomenon [6,7,8]. The process of projectile penetration into concrete exhibits extremely high transient complexity, encompassing high-strain-rate effects, dual nonlinearity of geometry and material, and multi-physics coupling characteristics [9,10,11]. Its failure mechanism is regulated by the macroscopic strength of the target, but essentially depends on the multi-phase component properties and complex interface evolution laws at the mesoscale [12,13,14]. Concrete can be regarded as a three-phase heterogeneous composite material comprising a random aggregate, a mortar matrix, and an interfacial transition zone (ITZ). The geometric morphology, particle size gradation, volume fraction, and spatial topological distribution of aggregate collectively determine the dynamic mechanical response characteristics and stress wave propagation path of the target, and further affect its anti-penetration performance [15,16]. Conventional homogenization modeling methods ignore the randomness and heterogeneity of the mesoscale components of concrete, and cannot accurately capture localized failure characteristics such as aggregate crushing, interface debonding, and mortar cracking, which limits the accuracy of numerical prediction [17].

With the development of numerical simulation technology, mesoscale modeling has become an important approach for investigating the penetration resistance of concrete [18,19,20]. By constructing mesoscale models that explicitly incorporate aggregate, mortar matrix, and ITZ, researchers can capture and characterize the internal damage evolution process and crack propagation path of materials under penetration impact, thereby providing critical support for the optimization design of macroscopic penetration resistance. Currently, the core challenges in mesoscale model construction mainly focus on three aspects: First, the geometrically realistic representation of the irregular characteristics of natural aggregates and the accurate reconstruction of their spatial random distribution [21,22,23]. Second, the efficient random placement of aggregates in the target, and avoiding numerical calculation distortion caused by local aggregate aggregation or spatial interference [20,24,25]. Third, the physical calibration of constitutive parameters for the three-phase components to ensure that the mesoscale model can accurately map the real meso-mechanical properties of concrete [26]. Compared with existing studies, the mesoscale model proposed in this paper can continuously generate the full-spectrum morphology of aggregate ranging from rounded pebbles to angular crushed stones. By predefining the background grid and strictly adopting a 50% grid area ratio as the criterion for determining intrusion and interface phase generation, the placement efficiency of polygonal aggregates is significantly improved. In this paper, a dynamic enhancement coefficient and a truncation mechanism are introduced into the concrete damaged plasticity model, which numerically guarantees the physical authenticity and computational stability of energy dissipation evolution in the projectile–target contact area. In addition, the coupled competition mechanism between projectile geometric/motion parameters and target meso-structure parameters and its influence law on damage patterns still lacks systematic and in-depth investigation at present.

To address this, the present study investigates the mesoscale dynamic behavior of high-strength concrete penetrated by rigid projectiles, and systematically carries out research on the penetration mechanism. First, by combining the random convex polygon algorithm and the background mesh mapping technique, a three-phase mesoscale numerical model that can truly reflect aggregate morphology and achieve uniform aggregate distribution is constructed. Second, material parameter calibration is performed by comparing with experimental data, and mesh sensitivity analysis is conducted to ensure the accuracy and convergence of the numerical solution. Subsequently, through large-scale parametric analysis, the effects of aggregate morphology, aggregate volume fraction, as well as projectile velocity, length-to-diameter ratio, and CRH on concrete damage evolution and projectile motion characteristics are systematically revealed. The conclusions are intended to provide a numerical calculation basis for the mesoscale optimization design of protective structures and the evaluation of their impact resistance performance.

This study establishes a three-phase mesoscale concrete simulation system that accounts for the strain-rate effect, breaking through the limitations of traditional macroscopic empirical formulas. The model adopts random convex polygon generation and background grid mapping technology, which truly restores the geometric morphology of aggregate and realizes automatic uniform placement, while implementing rate-dependent improvement on the plastic damage model. The regulation law of aggregate and projectile parameters on damage can optimize mixture proportioning and reduce experimental costs. The proposed model captures the asymmetric damage network induced by aggregate morphology and its amplification effect on projectile deflection. The obtained mechanism and quantitative conclusions support the impact-resistant design and assessment of protective structures, and can provide a reference for the revision of engineering specifications.

## 2. Concrete Mesoscopic Model Construction and Parameter Calibration

### 2.1. Generation of Random Convex Polygonal Aggregate Grains

**Figure 1:**
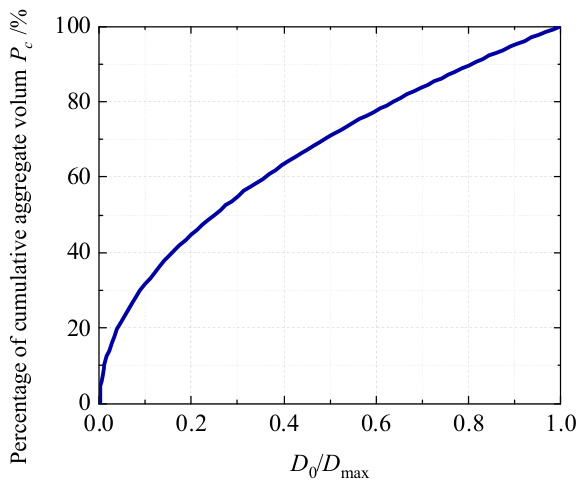
Fuller gradation curve [29].

The maximum density gradation curve presented in Figure 1 is the Fuller curve, which is applicable to three-dimensional aggregate gradation design. Concrete with this gradation can achieve the optimal matching of compactness and strength [27]. In view of the excessively high computational cost of three-dimensional mesoscale numerical concrete models, probability and statistical methods are often used to transform such models into two-dimensional planar models for analysis. Walraven and Reinhardt [28] established a correlation model between the volume fraction of aggregates and the area fraction of section aggregates in concrete specimens, realizing the conversion from the Fuller gradation curve to the probability distribution of aggregate particle size on two-dimensional sections.

The particle size distribution of aggregates is governed by the Fuller maximum density grading curve, which was originally established for three-dimensional aggregate systems [27]. Given the prohibitive computational expense of full 3D mesoscale simulations, a probabilistic transformation is commonly employed to reduce the problem to a two-dimensional representation. Walraven and Reinhardt [28] developed a statistical correlation linking the volumetric aggregate fraction in a concrete specimen to the corresponding areal fraction on a random cross-section, thereby enabling the conversion of the Fuller curve into a 2D aggregate size probability distribution, as expressed in Equation (1).

(1) $$P_{c}\left(D<D_{0}\right)=P_{k}\left(1.065{\left(\frac{D_{0}}{D_{\max}}\right)}^{0.5}-0.053{\left(\frac{D_{0}}{D_{\max}}\right)}^{4}-0.012{\left(\frac{D_{0}}{D_{\max}}\right)}^{6}-0.0045{\left(\frac{D_{0}}{D_{\max}}\right)}^{8}+0.0025{\left(\frac{D_{0}}{D_{\max}}\right)}^{10}\right)\;$$

where *P*_k_ denotes the total aggregate volume fraction, *D*_0_ represents a specific sieve size, and *P*_c_ is the probability of encountering aggregates smaller than *D*_0_ on a 2D section. This probability distribution is adopted in the present work to assign particle sizes to individual aggregates.

The target specimen in this study measures 1200 mm × 1200 mm. In the engineering field, the maximum nominal particle size of coarse aggregate is generally controlled between 20 mm and 31.5 mm [30,31]. When aggregates with relatively small particle sizes are adopted, concrete can be regarded as a homogeneous material [32,33]. In this study, the diameter of the projectile is set to 30 mm, and the ratio of the maximum aggregate particle size to the projectile diameter falls within the range of 0.5 to 1.5 [32]. For a two-dimensional planar model, the maximum aggregate particle size should not exceed one-third of the sample dimension. Based on the above principles, the aggregate particle size is determined as 10 mm to 30 mm in this paper. To enhance placement efficiency and minimize interference checks, aggregates are generated in descending order of size, ensuring compliance with the gradation specified by Equation (1).

**Figure 2:**
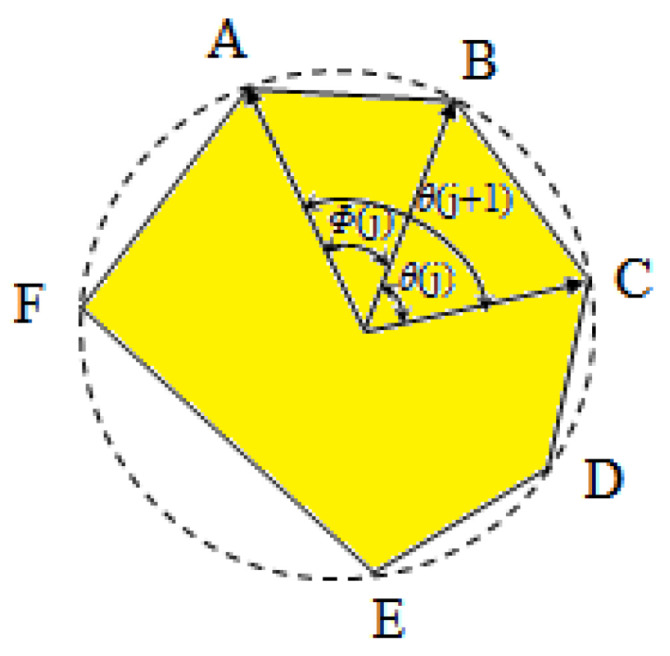
Schematic diagram of convex polygonal aggregate grain generated by inscribing in a circle [29].

This work develops an innovative algorithm to generate irregular convex aggregate particles circumscribed inside circular contours. Multiple vertex coordinates are randomly sampled along a circular perimeter and linked end-to-end to construct closed convex polygonal outlines, with each vertex located via its polar coordinate angle *θ*(*j*). It is seen in Figure 2.

(2) $$\Phi \left(j\right)=\frac{2\pi }{n}+\left(2\mathrm{rand}-1\right)\delta \cdot \frac{2\pi }{n}$$

The central angle between two adjacent vertices is defined as *Φ*(*j*) = *θ*(*j* + 1) − *θ*(*j*). For an n-sided polygonal particle, the theoretical average central angle per edge is fixed at 2π/n. The perturbed central angle can be expressed as:

To enforce the closure condition ∑*Φ*(*j*) = 2π, a normalization is applied:

(3) $$\bar{\Phi }\left(j\right)=\Phi \left(j\right)\cdot \frac{2\pi }{\sum_{i=1}^{n}\Phi \left(i\right)}$$

The final polar angle of each vertex is then computed cumulatively:

(4) $$\theta \left(j\right)=a+\sum_{i=1}^{j-1}\bar{\Phi }\left(i\right)$$

where rand is a uniformly distributed random number in [0, 1], and *δ* is a fluctuation coefficient controlling the angular deviation from the mean value 2π/n. *a* is an initial orientation angle, uniformly distributed over [0,2π]. The parameter *δ* ranges from 0 to 1. A higher value produces more angular and irregular particle shapes. Ultimately, this results in a significant reduction in the penetration depth of the projectile and an accelerated velocity attenuation rate. Based on prior microstructure modeling studies [34,35], a value of *δ* = 0.5 is adopted as a balanced representation of typical crushed aggregates. *j* is the vertex number of the convex polygonal aggregate. The characteristic size of each aggregate is defined as the maximum distance between any two vertices, and the count of aggregates in each size fraction is determined by comparing these sizes against the prescribed gradation intervals.

### 2.2. Random Aggregate Placement Method Based on Background Meshes

**Figure 3:**
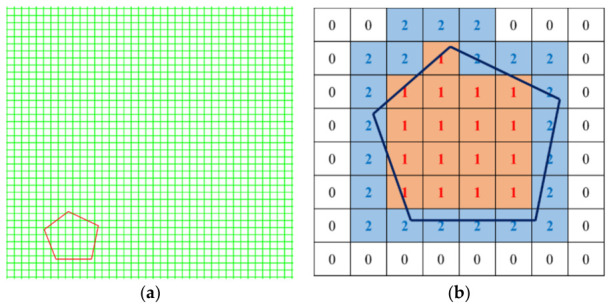
Background mesh and aggregate placement rules [29]: (**a**) background mesh; (**b**) property judgment for aggregate placement.

A background-grid-assisted placement strategy is developed to construct the three-phase mesoscale model efficiently. The procedure is implemented via a Python version 3.9 script interfacing with ABAQUS (version 2022) and comprises the following key steps:

1. Grid Generation and Initialization: A square domain of side length L is discretized into an n × n regular grid, serving as the background mesh, which can be seen in Figure 3. The mesh size is chosen to be approximately 1/5 to 1/10 of the minimum aggregate diameter [36]. Each element is assigned a property flag: 0 for mortar, 1 for aggregate, and 2 for ITZ, with all elements initially set to 0.
2. Aggregate Placement Protocol: Aggregate grains are placed sequentially from largest to smallest. For each new aggregate, a candidate centroid is randomly selected among elements with property 0, with the constraint that the entire polygon must lie within the domain and not intersect the outermost layer of elements. This restriction avoids artificial stress concentrations at the loading boundaries. Upon successful placement, all elements fully enclosed by the polygon are flagged as 1 (aggregate). For boundary elements partially covered by the aggregate, the flag is set to 1 if the covered area exceeds 50% of the element area; otherwise, it is assigned to 2 (ITZ).
3. Interference Detection: For each new candidate aggregate, a two-stage intrusion check is performed against all previously placed grains. First, a rapid pre-screening based on element-wise spatial proximity identifies potentially overlapping regions. Second, a precise geometric Boolean operation is conducted on the candidate elements to compute the actual intersection area. An aggregate is considered intrusive only if the intersection area exceeds half the element area, thereby avoiding false positives from minor overlaps.

4. ITZ Generation: Following the placement of all aggregates, a 2 mm thick ITZ layer is generated along the aggregate boundaries by assigning a property flag of 2 to adjacent elements. In the background grid method, an equivalent numerical thickness consistent with the basic grid size is generally adopted to characterize the interfacial transition zone [37,38]. A slight increase in the ITZ thickness will slightly expand the damage distribution range in the interface region, and promote more crack initiation along the periphery of the aggregate, yet it will not alter the macroscopic pattern. This equivalent thickness of 2 mm of ITZ is consistent with values reported in the literature [39,40]. Adjacent contacting aggregates may share the same ITZ region. This overlapping effect expands the scope of local weak zones, making it easier for stress concentration to release at the connected interfacial transition zones and enhancing the propagation continuity of cracks along weak interfaces.
5. Termination Criterion: The placement process terminates once the cumulative area of placed aggregates reaches the prescribed volume fraction for each gradation interval.

### 2.3. Incorporation of Rate Effect in the CDP Model

The nonlinear mechanical responses of the aggregate, mortar, and ITZ are described using the concrete damaged plasticity (CDP) model available in ABAQUS version 2022 [41,42]. Within the framework of continuum damage mechanics, the relationship between the nominal stress tensor *σ* and the effective stress tensor $\bar{\sigma }$ is expressed as:

(5) $$\sigma =\left(1-d\right)\bar{\sigma }=\left(1-d\right)D_{0}^{el}:\left(\varepsilon -\varepsilon^{pl}\right)$$

where *σ* and $\bar{\sigma }$ represent the stress and effective stress, respectively. *ε* and *ε*^pl^ denote the strain and plastic strain, respectively. $D_{0}^{el}$ is the initial stiffness, and *d* is the damage variable (Comprising compressive damage *d*_c_ and tensile damage *d*_t_). The variable “*d*” physically represents the degradation ratio of the effective elastic stiffness due to microcrack nucleation and propagation.

The penetration of a projectile into a target constitutes a typical working condition with intense dynamic loading, where the material is subjected to high-strain-rate conditions, and concrete exhibits significant rate sensitivity [14,43]. To accurately capture the dynamic enhancement effect, a dynamic increase factor (DIF) is introduced to correct the static strength. In the material definition, the rate-dependent options are activated through the concrete compression hardening and concrete tension stiffening options. Based on the CEB-FIP code [44] and the SHPB dynamic tests [45], namely Equations (6) and (7), the strain-rate hardening curves for compression and tension are constructed, respectively, by using the calibrated dynamic increase factors. A segmented power-law function is adopted to distinguish the strength enhancement laws in low-, medium-, and high-strain-rate intervals. Meanwhile, a correction term for dynamic peak strain is introduced to simultaneously match the nonlinear evolution of material deformability under high strain rates. An upper-bound truncation mechanism for dynamic increase factors is also established to avoid the non-physical infinite amplification of strength in the region near the projectile nose under GPa-level high pressure and ultra-high strain rates of 10^3^~10^5^ s^−1^. During the explicit analysis in ABAQUS, linear interpolation of the tabular data is automatically performed according to the equivalent strain rate of each element in the current increment step. This experiment-fitting-based rate-dependent model can well describe the strength enhancement law of concrete from low strain rate to high strain rate under impact loading, which lays a theoretical foundation for in-depth analysis of the failure mechanism during penetration.

The calculation equation for the dynamic increase factor of concrete compressive strength is given as follows [45]:

(6) $$DIF_{c}=\frac{\sigma_{cd}}{\sigma_{cs}}=\left\{\begin{array}{ll} 1 & \dot{\varepsilon }\leq {\dot{\varepsilon }}_{stat}\\ {\left(\frac{\dot{\varepsilon }}{{\dot{\varepsilon }}_{stat}}\right)}^{1.026\alpha } & {\dot{\varepsilon }}_{stat}<\dot{\varepsilon }\leq 30{\;s}^{-1}\\ \gamma {\left(\dot{\varepsilon }\right)}^{\frac{1}{3}} & \dot{\varepsilon }>30{\;s}^{-1} \end{array}\right.$$

where *σ*_cd_ is the dynamic compressive strength, *σ*_cs_ is the static compressive strength,$\;{\dot{\varepsilon }}_{stat}$ is the quasi-static strain rate, which is taken as 3 × 10^−5^ s^−1^. lgγ = 6.156α − 0.49, α = (5 + 3*σ*_cu_/4)^−1^ = (5 + 9*σ*_cs_/*σ*_c0_)^−1^, *σ*_cu_ is the compressive strength of cubic concrete (in MPa), and *σ*_c0_ = 10 MPa is the reference value.

The equation for calculating the dynamic increase factor of concrete tensile strength is given by [45]:

(7) $$DIF_{t}=\frac{f_{td}}{f_{ts}}=\left\{\begin{array}{ll} {\left(\frac{\dot{\varepsilon }}{{\dot{\varepsilon }}_{0}}\right)}^{1.016\delta } & {\dot{\varepsilon }}_{stat}<\dot{\varepsilon }\leq 30{\;s}^{-1}\\ \eta {\left(\frac{\dot{\varepsilon }}{{\dot{\varepsilon }}_{stat}}\right)}^{\frac{1}{3}} & \dot{\varepsilon }>30{\;s}^{-1} \end{array}\right.$$

where *f*_td_ is the dynamic tensile strength. *σ*_cs_ is the static tensile strength.$\;{\dot{\varepsilon }}_{stat}$ is the quasi-static strain rate, which is taken as 3 × 10^−6^ s^−1^. lgη = 7.11δ – 2.33, *δ* = 1/(10 + 6*f*_cs_*f*_0_^−1^). *f*_cs_ is the compressive strength of cubic concrete (in MPa), and *σ*_c0_ = 10 MPa is the reference value.

Since the standard CDP model built into ABAQUS version 2022 is based on the static constitutive relationship by default, it cannot directly represent the complex coupling logic between damage evolution and strain rate. In this study, the secondary development and improvement of the CDP model are achieved by constructing a series of rate-dependent uniaxial stress–strain hardening curves. In this process, the dynamic constitutive curve is not simply linearly scaled. Instead, the non-linear mapping of both strength enhancement and deformation capacity evolution is considered. Specifically, as the strain rate increases, the dynamic stress value increases in proportion to the dynamic increase factor (DIF), and the corresponding peak strain also shifts synchronously, as shown in Equation (8). Thus, it fully depicts the physical characteristics of the dual enhancement of strength and toughness of concrete under high strain rates.

Regarding the evolution of the dynamic peak strain, this study follows the power–law mapping relationship recommended by the CEB-FIP code and relevant dynamic mechanical experimental studies, and its expression is as follows:

(8) $$\varepsilon_{c,dyn}=\varepsilon_{c\theta }{\left(\frac{\dot{\varepsilon }}{{\dot{\varepsilon }}_{\theta }}\right)}^{n_{c}}$$

where *ε*_c,dyn_ and *ε*_cθ_ denote the peak compressive strain under dynamic and quasi-static conditions, respectively. ${\dot{\varepsilon }}_{\theta }$ is the quasi-static reference strain rate, and the exponent *n*_c_ is set to 0.02. Similarly, the evolution of peak dynamic tensile strain *ε*_t,dyn_ follows the same logic, and its power-law exponent *n*_t_ is generally calibrated to 0.02–0.03 based on experimental data.

Considering the numerical singularity caused by extremely high strain rates that may occur in local elements during penetration calculations, this study further introduces a DIF truncation mechanism. In the classical review published by Malvar and Crawford [45] in 1998, it is pointed out that the dynamic increase factor of concrete can exceed 2 under compression and exceed 6 under tension [46,47,48]. In this paper, the cut-off value of compressive DIF is set to 3.0, and that of tensile DIF is set to 6.0. This selection is based on the possible upper magnitude limit of DIF reported in the aforementioned literature. This improvement not only ensures the effective activation of dynamic strengthening effects under high strain rates, but also eliminates the risk of numerical overflow. In the built-in rate-dependent table function of the ABAQUS/CDP model, after inputting the corresponding dynamic strength values for different strain rates, the strength response of the model at each strain rate is automatically interpolated by ABAQUS.

### 2.4. Calibration of Mesoscopic Model Calculation Parameters

**Figure 4:**
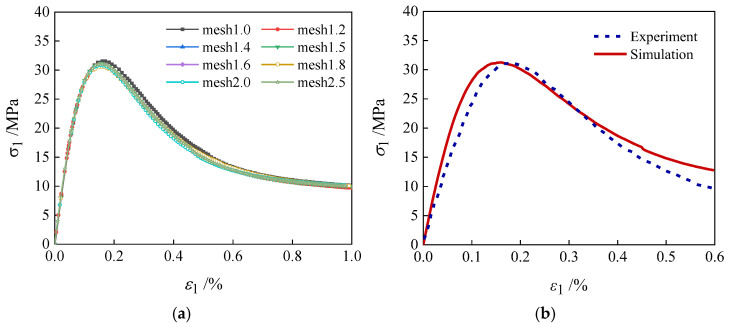
Uniaxial compression stress–strain curves from numerical simulations and experimental tests [29]: (**a**) stress–strain; (**b**) comparison between simulation and experiment.

To verify the rationality and accuracy of the calculation parameters and mesh division of the established concrete mesoscale model, a mesh sensitivity analysis is performed on 8 meshes with different element lengths (Le), namely 1.0 mm, 1.2 mm, 1.4 mm, 1.5 mm, 1.6 mm, 1.8 mm, 2.0 mm, and 2.5 mm. Figure 4 presents a comparison between the simulated uniaxial compressive stress–strain response and the experimental data reported by Shang [49]. The numerical curve shows excellent agreement with the experimental results, particularly in the elastic ascending branch and the hardening phase, with the peak stress error maintained within 10%. This validation confirms the reliability of the assigned material properties for the three phases.

As a thin interfacial layer in concrete, the ITZ is determined by adopting the mortar strength reduction method, with its strength ranging from 50% to 90% of that of bulk mortar [50,51,52]. In this study, a reduction coefficient of 83% is ultimately selected. The equation for calculating the shear strength of the mortar matrix is *τ*_m_ = 0.1*f*_cm_, where *τ*_m_ denotes the shear strength of the mortar and fcm represents the static compressive strength of the mortar. The shear strength of the interfacial transition zone (ITZ) is 83% of the shear strength of mortar. The plastic damage parameters of mortar are determined based on the recommended values of the concrete damaged plasticity (CDP) model in ABAQUS [53] and calibrated through experimental tests. The CDP model is adopted for concrete aggregates, mortar, and the interfacial transition zone, and the key parameters of the model are presented in Table 1.

**Figure 5:**
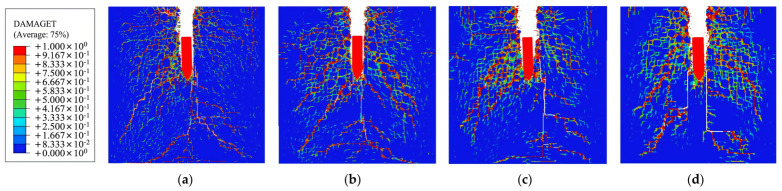
Distribution map of concrete tensile damage under different mesh sizes (t = 0.0005 s): (**a**) mesh = 1.0 mm; (**b**) mesh = 1.5 mm; (**c**) mesh = 2.0 mm; (**d**) mesh = 2.5 mm.

**Figure 6:**
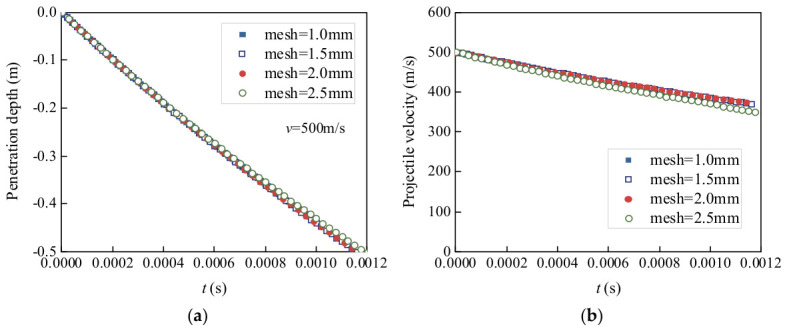
Comparison chart of penetration depth and velocity attenuation law under different mesh sizes: (**a**) penetration depth; (**b**) velocity attenuation law.

The uniaxial compression test of concrete verifies that the parameters of the CDP model for aggregate, mortar, and ITZ adopted in this study are reasonable and reliable. On this basis, the above parameters are applied to the numerical simulation of projectile penetration into concrete targets. The strain-rate effect is introduced into the CDP model to characterize the dynamic loading characteristics. To assess the influence of mesh resolution on penetration predictions, simulations are performed with element sizes of 1.0, 1.5, 2.0, and 2.5 mm (Figure 5 and Figure 6). While the detailed damage morphology exhibits some mesh dependency—as cracks preferentially propagate along element boundaries— the global measures of penetration depth and projectile velocity decay are found to be largely insensitive to mesh size. Considering the trade-off between computational cost and accuracy, a uniform mesh size of 2.0 mm is adopted for all subsequent penetration simulations, consistent with previous mesoscale studies [39,40].

Prior to conducting large-scale parametric penetration simulations, this study systematically carried out mesh sensitivity tests under dynamic loading conditions. Although material damage generally propagates along mesh boundaries, resulting in slight differences in the morphology of local damage distribution under different background mesh sizes, the overall penetration depth and the attenuation law of projectile velocity exhibit extremely low dependence on mesh size. For the validation of high-strain-rate-related parameters, the calculation of the dynamic increase factor (DIF) of concrete compressive strength directly adopts the formula recommended by the CEB-FIP code and the fitting parameters obtained from SHPB tests. These formulas and parameters have already been sufficiently validated based on extensive experimental data.

Regarding the treatment of extreme local mesh distortion in the ballistic tunnel region, this study adopts the mesh failure and deletion criterion. When the equivalent plastic strain or damage variable at the internal integration point of an element reaches the preset critical threshold, the element is automatically removed from the global calculation matrix, and the stress it carries is subsequently reset to zero, thereby forming a macroscopic penetration cavity. A lower penetration threshold leads to easier element deletion, reduces the resistance exerted on the projectile, and results in a larger calculated penetration depth. The element deletion method inevitably introduces artificial losses of mass, momentum, and energy, which is an inherent drawback of this approach. In this paper, dc = 0.95 is set as the complete damage threshold, which exactly corresponds to the critical plastic deformation state when the material completely loses its load-bearing capacity. Only fractured elements that no longer provide effective load-bearing capacity are removed, while intact matrix, aggregate, and interface elements that still maintain force transmission capacity are not eliminated prematurely, which substantially reduces the spurious energy dissipation caused by artificial erosion.

### 2.5. Modeling of Concrete Target and Rigid Projectile

**Figure 7:**
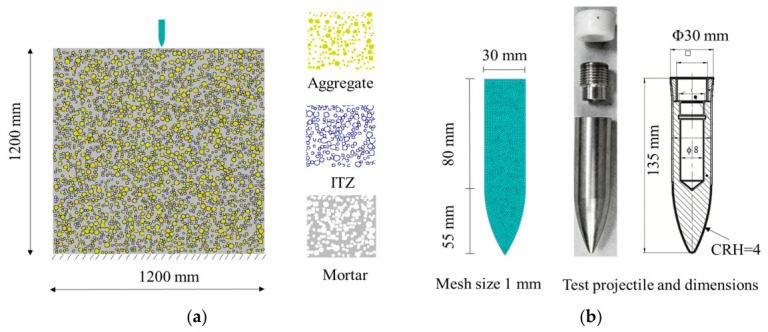
Mesoscopic model of three-phase medium of concrete target and rigid projectile: (**a**) concrete target; (**b**) rigid projectile.

Figure 7 presents the geometric model of the concrete target and the projectile. The dimension of the concrete target is set to 1200 mm × 1200 mm, which is consistent with the models adopted in relevant experiments and existing literature [54]. In the numerical model, fixed constraints are applied to the bottom of the target, while the side edges and the upper boundary are set as free boundaries [55]. To eliminate the influences of boundary effect and size effect, this study follows the general industrial standard to set the ratio of the target width to the projectile diameter to be no less than 15–20, so as to ensure that the target can be equivalent to a semi-infinite medium. The diameter of the projectile is preliminarily determined to be 25–33 mm. Meanwhile, considering that the aggregate particle size in the mesoscale model of this study ranges from 10 mm to 30 mm, the ratio of the maximum aggregate particle size to the projectile diameter is controlled between 0.5 and 1.5 to ensure that the projectile acts simultaneously on the aggregate, mortar, and ITZ during penetration. For conventional rigid projectiles, the length–diameter ratio L/D is generally set as 3–5. In this study, the size and shape of the projectile are consistent with those of the actual projectile used in the experiment [56]. The projectile diameter is 30 mm, the projectile length is 135 mm, and the caliber-radius-head CRH is 4. All the parameters satisfy the above constraint conditions.

The adoption of a two-dimensional planar model in this paper is primarily motivated by considerations of computational efficiency for large-scale parametric analysis, yet it nonetheless exhibits inherent limitations. Specifically, the two-dimensional model underestimates the overall propagation range of cracks and the complexity of spatial interlacing to a certain extent, completely restricts the self-rotation of the projectile around its axis, and neglects the stacking in the aggregate thickness direction and the interlayer interlocking effect. The proposed model is more suitable for qualitatively revealing the influence law of each variable on damage evolution and penetration depth, and focuses on trend analysis at the mechanism level. To achieve accurate quantitative prediction of live projectile penetration results, a three-dimensional mesoscale model needs to be constructed in subsequent research to eliminate the deviation introduced by the planar assumption.

## 3. Results and Discussions

### 3.1. Effects of Aggregate Shape and Aggregate Volume Fraction on the Penetration Process

**Figure 8:**
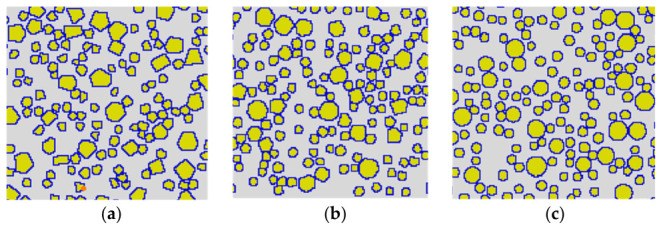
Schematic diagram of specimens with different aggregate geometries: (**a**) polygonal specimen 1; (**b**) polygonal specimen 2; (**c**) circular specimen.

In practical reinforced concrete structures, coarse aggregates typically exhibit polyhedral or ellipsoidal morphologies [57]. To better represent the mesoscale heterogeneity of concrete, three distinct aggregate geometries are considered in this study: Polygon 1 (with 4–6 edges per grain), Polygon 2 (with 5–8 edges), and circular aggregates. These three shape types are schematically shown in Figure 8, with a nominal aggregate volume fraction of approximately 45% for all specimens. Unless otherwise specified, Polygon 1 is adopted as the baseline configuration for the parametric analyses.

**Figure 9:**
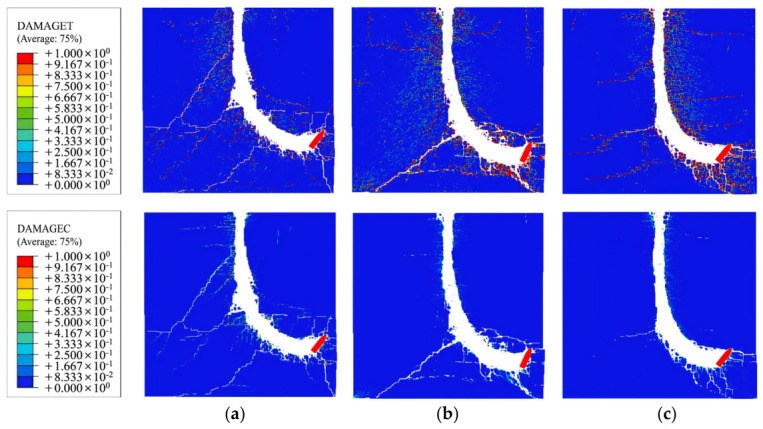
Schematic diagram of tensile damage and compressive damage of three different sample specimens with different aggregate shapes after the projectile velocity decayed to 0: (**a**) polygonal specimen 1; (**b**) polygonal specimen 2; (**c**) circular specimen.

Figure 9 presents the spatial distribution contours of tensile damage (DAMAGET) and compressive damage (DAMAGEC) for three shapes of aggregate specimens at t = 0.01 s. The damage value ranges from 0 to 1, where blue indicates no damage (0), and red represents complete damage/failure (1), with higher values corresponding to more severe damage. Once the equivalent strain or stress within an element (representing mortar, aggregate, or ITZ) reaches the preset failure threshold, the element will be removed from the calculation.

In terms of tensile damage, in polygonal specimen 1, the main crack path exhibits obvious zigzag deflection and bifurcation characteristics. A large number of secondary cracks propagate along the aggregate interface, forming a large-range, high-density multi-branched crack network. The high-damage zone is not only concentrated on the wall of the penetration channel, but also extends to the deep and lateral parts of the target. In polygonal specimen 2, the crack deflection and branching effects are slightly weakened. Clear aggregate-induced secondary cracks are still retained, and the crack network density and damage range are between those of crushed stone aggregate and circular aggregate. For circular specimens, the cracks are dominated by smoothly penetrating main cracks, with almost no aggregate-induced branched cracks, and the crack path is single. The high tensile damage zone is only confined to the vicinity of the penetration channel. The overall coverage of tensile damage to the target is significantly smaller than that of polygonal aggregate specimens.

In terms of compressive damage, the compressive damage zones of polygonal specimens 1 and 2 exhibit complex morphology. The crushed zone is distributed along the edges and interfaces of aggregates, forming local high compressive damage zones at the aggregate-mortar interface, with a larger depth and lateral extension range of the crushed zone. The boundary of the crushed zone of polygonal specimen 1 presents a multi-peak shape, and shear-type crushed zones are more developed. The crushing zone boundary of polygonal specimen 2 is relatively smooth, but an obvious interfacial compressive damage enhancement zone still exists. For circular aggregate specimens, the high compressive damage zone is only concentrated on the channel wall directly in front of the projectile. The extent of the comminuted zone is minimized, with negligible compressive damage in the matrix outside the channel.

Overall, the sharper the aggregate edges and corners, the stronger the interface-induced crack deflection and branching effects, leading to a wider propagation range of tensile and compressive damage in the target and more complex damage modes. In contrast, for circular aggregates, the interfacial stress is uniformly distributed, the crack propagation path remains undisturbed, damage is concentrated in local channel areas, and the overall damage degree of the target is lower.

In Figure 9, the percentage of damaged area can be adopted as the quantitative indicator. In the post-processing of ABAQUS, the proportion of the area of elements where the damage variables DAMAGET and DAMAGEC exceed 0.1 in the total target area is statistically calculated. According to rough estimation, the tensile damage area of the polygonal aggregate specimen 1 is approximately 1.5 to 2 times that of the circular aggregate specimen, and the compressive damage area is approximately 1.3 to 1.6 times that of the latter.

**Figure 10:**
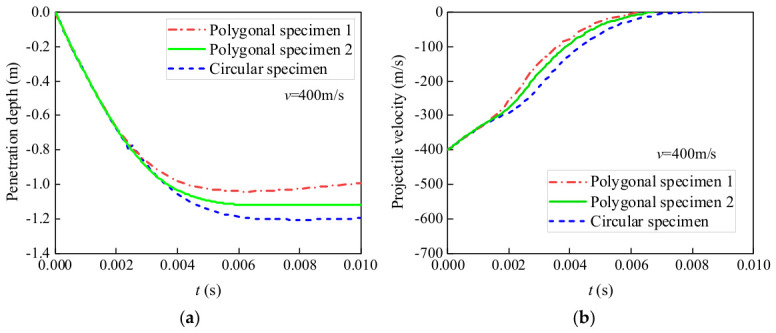
Effect of aggregate shape on the penetration depth and velocity attenuation law: (**a**) penetration depth; (**b**) velocity attenuation.

Figure 10 presents the curves of penetration depth and projectile velocity for three samples with different aggregate shapes under an initial projectile velocity of 400 m/s. Figure 10a shows that at the initial penetration stage (penetration depth = 0.7 m, t = 0.0021 s), the penetration depth growth rates of the three samples are basically consistent. As time elapses, the influence of aggregate shape shows differences. The more regular the aggregate shape is, the greater the increase in penetration depth is. The final penetration depth of the circular specimen is approximately 18% higher than that of the polygonal specimen 1, which is attributed to the fact that angular, sharp crushed aggregates can significantly restrict the projectile penetration process. Figure 10b illustrates that at the initial penetration stage (t = 0.0013 s), the velocity attenuation curves of the three types of aggregates basically overlap. At this moment, the projectile velocity is 320 m/s, which represents a 20% reduction compared with the initial velocity. In the middle and late penetration stages, the projectile velocity attenuates the fastest in polygonal specimen 1, while the attenuation is the gentlest in the circular specimen. This difference originates from the regulation of aggregate shape on interfacial stress transfer and energy dissipation paths. Polygonal aggregates substantially increase penetration resistance and accelerate projectile energy dissipation by inducing interfacial stress concentration and forming complex crack propagation paths. In contrast, interfacial stress distributes uniformly around circular aggregates, with a smooth crack propagation path, resulting in lower resistance of the target to the projectile. Consequently, a larger penetration depth and slower projectile velocity attenuation are observed.

The effect of random aggregate distribution on penetration results under a fixed particle shape is not discussed in this paper. This is because a large number of existing research findings have confirmed that random aggregate distribution has little influence on the statistical mean of penetration depth, but it significantly increases the statistical deviation (standard deviation) and exerts a crucial impact on the damage zone [20,58]. The results presented in this paper are caused by aggregate angularity, rather than the variability induced by random distribution [59]. The influence of random arrangement is localized and limited. The meso-structure of aggregates has little effect on core indicators such as penetration depth, and its main influence is reflected in local asymmetric effects such as ballistic deflection [20,60]. Aggregate angularity dominates the macroscopic response. The magnitude of the response difference induced by changes in aggregate angularity is far larger than the numerical dispersion caused by random aggregate arrangement.

The kinetic energy dissipation of the projectile can be directly calculated based on the velocity attenuation curve shown in Figure 10, which is defined as the difference between the initial kinetic energy and the real-time kinetic energy. According to the data in Figure 10b, when the projectile velocity attenuates to the stable stage, the kinetic energy loss of the projectile corresponding to the polygonal aggregate specimen 1 is approximately 92% of the initial kinetic energy, while that corresponding to the circular aggregate specimen is approximately 85%, with a difference of approximately 7% between the two. This additional kinetic energy dissipation is mainly attributed to the interfacial fracture energy consumed by crack deflection and branching induced by angular aggregates.

**Figure 11:**
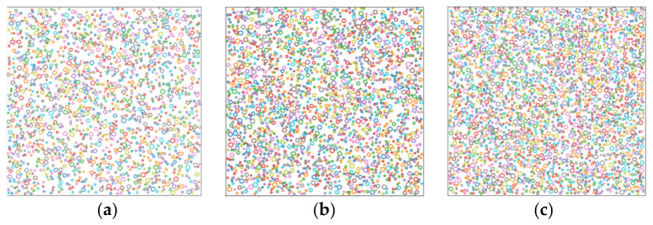
Schematic diagram of different aggregate volume fractions for polygonal specimens 1: (**a**) 35%; (**b**) 40%; (**c**) 45%.

**Figure 12:**
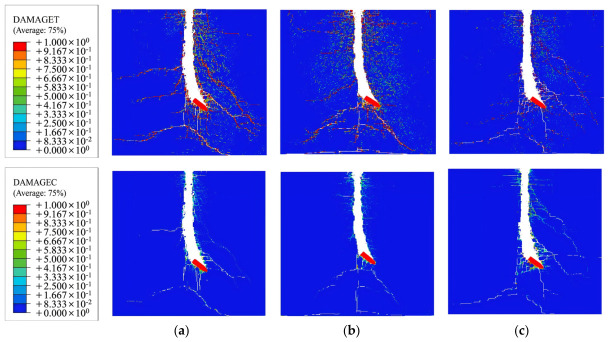
Effect of the volume fraction of aggregates on the tensile and compressive damage states of the concrete target: (**a**) 35%; (**b**) 40%; (**c**) 45%.

Figure 11 presents the schematic diagrams of concrete target models with aggregate volume fractions of 35%, 40%, and 45%, respectively. Figure 12 illustrates the distribution profiles of tensile damage (DAMAGET) and compressive damage (DAMAGEC) in concrete targets with different aggregate volume fractions at t = 2200 μs (before the projectile trajectory deflects horizontally) under penetration at a projectile velocity of 500 m/s. Under the specimen with an aggregate volume fraction of 35%, a large range tensile damage zone is formed around the projectile penetration path, where radial and oblique branch cracks are fully developed. The surface damage of the target exhibits a multidirectional divergence pattern. As the aggregate volume fraction increases from 35% to 45%, the scope of both the tensile and compressive damage zones in the target shows a significant shrinking trend. In particular, the number of radial cracks on the target surface and secondary microcracks around the projectile penetration path decreases remarkably. The inhibitory effect of high-proportion aggregates on crack propagation is enhanced. This finding is consistent with the conclusions of previous experiments. It should be noted that due to differences in aggregate type, matrix strength, projectile parameters, and experimental conditions, the numerical results obtained in this paper cannot be directly quantitatively aligned with experimental measurements.

**Figure 13:**
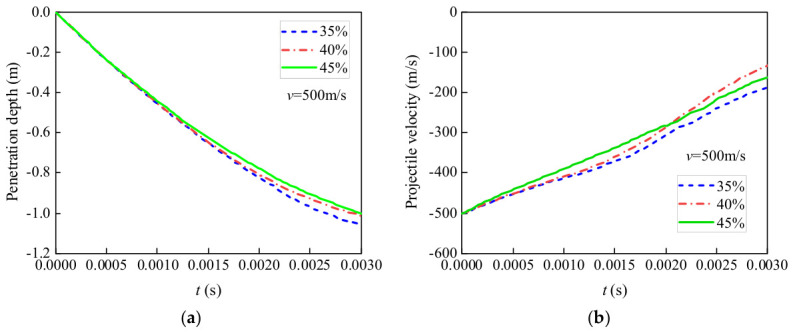
Effect of volume fraction of aggregates on the penetration depth and velocity attenuation law: (**a**) penetration depth; (**b**) velocity attenuation.

Figure 13 demonstrates that a higher aggregate volume fraction corresponds to a smaller penetration depth of the projectile and a faster velocity attenuation rate. The projectile penetration depth is the largest and the velocity attenuation is the slowest when the aggregate volume fraction is 35%. When the aggregate volume fraction reaches 45%, the penetration depth is significantly restricted, and the velocity decreases more drastically. These results indicate that high aggregate content significantly improves the penetration resistance of concrete targets. Through the direct blocking effect of aggregates on projectiles and the constraint of aggregates on crack propagation in the matrix, the energy dissipation and impact resistance of the target are enhanced. The crack extension and projectile penetration capability during the penetration process are effectively weakened.

### 3.2. Penetrating Mechanism

**Figure 14:**
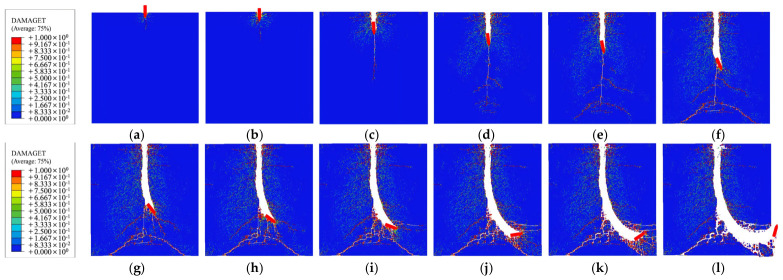
Evolution law of tensile damage during the penetration process of polygonal sample 1 (*v* = 500 m/s): (**a**) 100 µs; (**b**) 200 µs; (**c**) 500 µs; (**d**) 800 µs; (**e**) 1000 µs; (**f**) 1500 µs; (**g**) 2000 µs; (**h**) 2500 µs; (**i**) 3000 µs; (**j**) 4000 µs; (**k**) 5000 µs; (**l**) 10,000 µs.

**Figure 15:**
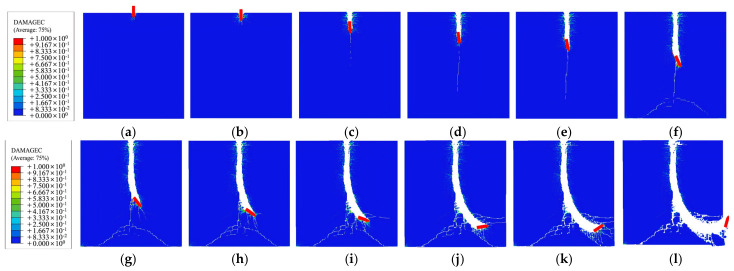
Evolution law of compressive damage during the penetration process of polygonal sample 1 (*v* = 500 m/s): (**a**) 100 µs; (**b**) 200 µs; (**c**) 500 µs; (**d**) 800 µs; (**e**) 1000 µs; (**f**) 1500 µs; (**g**) 2000 µs; (**h**) 2500 µs; (**i**) 3000 µs; (**j**) 4000 µs; (**k**) 5000 µs; (**l**) 10,000 µs.

Figure 14 and Figure 15 illustrate that during the entire process of the projectile penetrating the concrete target, the tensile and compressive damages of the target exhibit differentiated dynamic evolution characteristics. (a) In the initial stage of penetration (100–200 μs), both tensile and compressive damages first initiate at the contact point where the projectile hits the target, forming only local micro-damage areas with relatively low damage ranges and degrees. (b) As the penetration process progresses (500–1500 μs), the tensile damages (red and orange) rapidly extend into the interior of the target along the axis of the projectile. Meanwhile, radial microcracks with a nearly symmetric distribution are generated on the surface of the target. Transverse cracks gradually appear at the bottom of the target and expand to both sides. At 1500 μs, the damage extends to the bottom of the model. In contrast, the compressive damage is mainly concentrated in the penetration channel area in direct contact with the projectile, and their distribution range is relatively limited. (c) In the middle and later stages (2000–10,000 μs), the axial main cracks dominated by tensile damage continue to widen and connect with the transverse cracks at the bottom, forming a complete penetration channel. The microcracks on both sides of the channel and at the edge of the target further develop and converge. The compressive damage is always centered around the wall surface of the penetration channel. The high-value areas of compressive damage are always concentrated in the penetration channel area directly extruded by the projectile. The material on the channel wall surface first reaches a state of complete damage. The degree of compressive damage in the surrounding matrix is significantly lower than that of tensile damage, which intuitively reflects the differences in the stiffness degradation and failure paths of the target material in different stress-bearing areas during the penetration process. As the penetration depth increases, the compressive damage gradually expands slightly towards the bottom and periphery of the channel. This reflects the difference in the failure modes of the concrete target under impact loads, namely, “tensile-dominated cracking and compression-dominated compaction”.

The projectile orientation undergoes significant changes during the penetration process. (a) In the early penetration stage (100–1000 μs), the projectile nose moves stably along the initial axis, with no obvious change in orientation. (b) After entering the middle and late stages (after 1500 μs), affected by the propagation of asymmetric tensile cracks inside the target and the uneven distribution of material resistance, the projectile nose gradually deflects. The trajectory deviates from the direction of the initial axis, and the deflection angle continuously increases with the increase in penetration depth. (c) In the late penetration stage (5000–10,000 μs), the deflection trend of the projectile nose intensifies. Finally, an obvious asymmetric penetration channel is formed at the bottom of the target. Meanwhile, the asymmetry of the compressive damage distribution develops synchronously with the deflection of the projectile nose, which further intensifies the dynamic interaction between the projectile and the target. This reflects the significant disturbance effect of the asymmetric tensile and compressive damage of the concrete target on the projectile orientation and trajectory stability.

**Figure 16:**
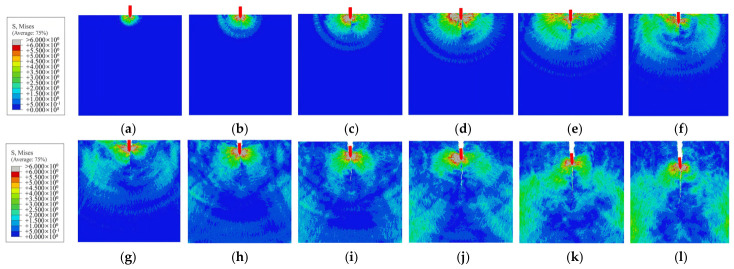
The stress wave propagation process of the polygonal specimen 1 (*v* = 500 m/s): (**a**) 40 µs; (**b**) 80 µs; (**c**) 120 µs; (**d**) 160 µs; (**e**) 200 µs; (**f**) 240 µs; (**g**) 280 µs; (**h**) 360 µs; (**i**) 400 µs; (**j**) 500 µs; (**k**) 600 µs; (**l**) 700 µs.

Figure 16 presents the stress propagation and damage evolution laws during the penetration of a rigid projectile into a concrete target, up to the stage where the first macroscopic crack appears (t = 1600 μs). (a) At the initial penetration stage, the compressive stress wave at the contact interface propagates radially from the contact point into the target interior. As the stress wave continuously diffuses, a large-scale compressive stress field is formed inside the target. The material beneath the contact interface enters the plastic yielding state because the stress exceeds the dynamic yield strength, and micro-damage initiates and propagates along the front of the stress wave. With the advancement of penetration, the material undergoes plastic yielding under high pressure and micro-damage nucleates. (b) Entering the middle and late stages of penetration, the stress wave propagates to the target boundary and reflects. The superposition of the incident compressive wave and the reflected tensile wave induces a tensile stress region inside the target, leading to the nucleation of radial microcracks and their rapid propagation towards the interior of the target. Meanwhile, under the action of high pressure and high strain rate, element deletion and fragmentation occur in the material near the penetration channel, forming the initial penetration cavity. (c) In the late penetration stage, the penetration channel is stably formed, and the target enters the stage of cratering and damage propagation. The material on the channel wall continuously spalls off, and cracks further coalesce to form a macroscopic fracture zone.

Section 2.4 verifies that the damage paths under different meshes differ slightly with mesh size, while the macroscopic characteristics and arrival timing of wavefront propagation are basically consistent across different meshes. This indirectly indicates that the stress wave propagation law is insensitive to mesh size. The stress wave propagation process shown in Figure 16 is intercepted up to 700 μs, during which the wavefront is still in the stage of propagating to the deep part of the target laterally, and has not yet reached the bottom and side boundaries of the target. Therefore, neither mesh size nor boundary reflection causes substantial interference to the penetration depth and velocity attenuation results concerned in this paper.

**Figure 17:**
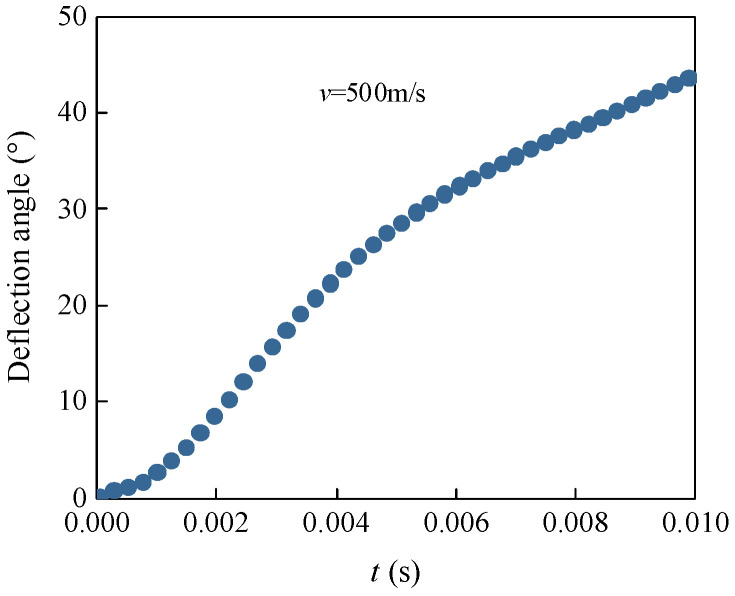
Variation in projectile deflection angle during penetration process.

By real-time monitoring of the displacement time-history data of the reference point on the projectile body, the displacement components of the projectile along the initial incident direction (Y-direction) and perpendicular to the incident direction (X-direction) can be extracted in post-processing, and the ballistic deflection angle can be calculated accordingly. Specifically, the tangent of the deflection angle is equal to the ratio of the X-direction displacement to the Y-direction displacement. The results are shown in Figure 17. The fundamental cause of projectile deflection is the deflection moment generated by asymmetric forces acting on the projectile during the penetration process. As illustrated in the figure, the projectile deflection angle increases monotonically with penetration time. The angle grows slowly in the initial stage of penetration; as the projectile warhead completely penetrates the target, the lateral overturning moment acts continuously, leading to a significant increase in the growth rate of the deflection angle. There is no ballistic correction and recovery stage throughout the process, and the deflection accumulates continuously during penetration.

### 3.3. Projectile Velocity

**Figure 18:**
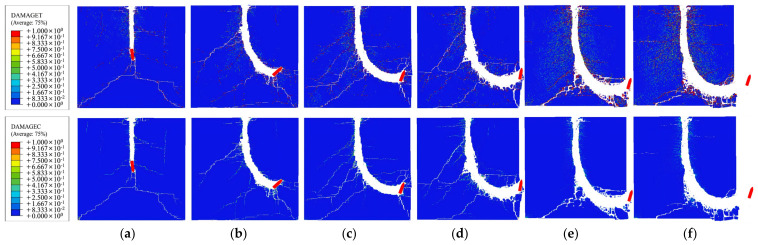
Effect of projectile velocity on the final distribution patterns of tensile and compressive damage of the concrete target body: (**a**) 100 m/s; (**b**)200 m/s; (**c**) 300 m/s; (**d**) 400 m/s; (**e**)500 m/s; (**f**) 600 m/s.

Figure 18 presents the nephograms of tensile and compressive damage of concrete targets under different initial projectile velocities (100–600 m/s). The projectile velocity exerts a significant influence on the damage evolution and failure mode during the penetration process. With the increase in initial velocity, both the penetration depth and crater diameter show a pronounced increasing trend. The crater morphology gradually transforms from a shallow, narrow circular hole at low velocities to a deep and wide funnel shape at high velocities. Tensile damage is mainly distributed along the penetration hole wall, the free surface of the target, and the propagation path of radial cracks. As the velocity increases, the damage range expands rapidly from the local contact area to the front half of the target. The crack network evolves from a single main crack into a multi-branch connected crack system. In contrast, compressive damage is concentrated in the contact area between the projectile and the target as well as the penetration hole wall. Under high-velocity conditions, a large-area compressive crushing zone forms at the front end of the target, which couples with tensile damage, leading to severe comminuted failure of the concrete near the projectile impact surface. On the whole, at low velocities, the target is dominated by local penetration and a small number of radial tensile cracks. At medium velocities, the coupling effect between tensile crack propagation and hole wall crushing is enhanced. At high velocities, the high-strain-rate effect dominates the failure process, forming a typical layered failure structure of “crushing zone–crack propagation zone–elastic zone”. The penetration efficiency increases significantly with the increase in velocity.

**Figure 19:**
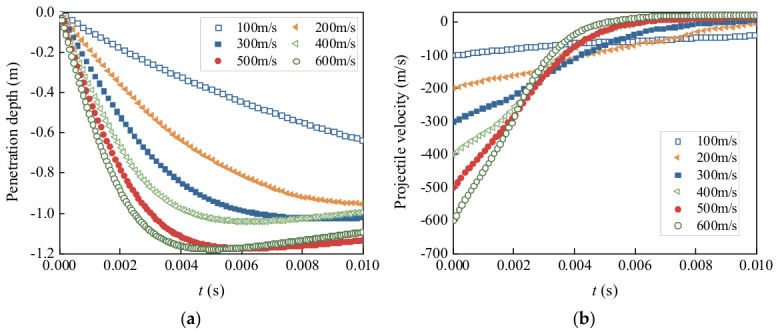
Effect of initial velocity of the projectile on the penetration depth and velocity attenuation law: (**a**) penetration depth; (**b**) velocity attenuation.

Figure 19 presents the evolution curves of penetration depth and projectile residual velocity versus time under different initial projectile velocities (100–600 m/s). A higher initial velocity leads to a greater ultimate penetration depth and a faster attenuation rate of projectile velocity. When the initial velocity is 100 m/s, the penetration depth increases linearly, the projectile velocity attenuates approximately linearly, and the projectile experiences almost no deflection. For other initial velocity conditions, the penetration depth increases linearly in the initial stage of penetration and then tends to stabilize, which is attributed to the deflection of the projectile from the vertical direction to the horizontal direction. For the conditions with initial velocities of 500 m/s and 600 m/s, the target is almost completely penetrated, which demonstrates the dynamic anti-penetration enhancement effect of concrete under high strain rates. The attenuation process of projectile velocity exhibits nonlinear characteristics. Under low velocity conditions, the velocity attenuates approximately linearly, and the residual velocity is relatively high. Under medium and high-velocity conditions, the velocity decreases sharply in the initial penetration stage, after which the attenuation rate decreases significantly and eventually stabilizes at a residual velocity, which increases as the initial velocity rises.

Taking the working condition of 500 m/s in this paper as an example, the local strain rate near the projectile–target interface can reach the order of 10^2^~10^3^ s^−1^, the corresponding compressive DIF is approximately 2.0~3.0, and the tensile DIF is approximately 4.0~6.0. This indicates that the actual dynamic strength of the material in the contact area is much higher than its static strength, and this strength increase is directly reflected in the increase in penetration resistance. The strain rate level varies with the initial velocity, leading to corresponding variations in DIF, which can quantitatively explain why more energy is consumed per unit penetration depth at higher velocities, that is, the dynamic strengthening effect. Second, quantitative analysis can be conducted from the perspective of energy dissipation. Based on the projectile velocity attenuation curve shown in Figure 19b, the total kinetic energy absorbed by the target and its distribution along the penetration depth under different initial velocities can be calculated. Taking the comparison between the working conditions of 400 m/s and 600 m/s as an example, the kinetic energy of the projectile at 400 m/s is calculated as 1/2 mv^2^, and the kinetic energy at 600 m/s is 2.25 times that of the former, while the ratio of the final penetration depth of the two is far less than 2.25. This demonstrates that the energy dissipation rate per unit penetration depth increases significantly under high-velocity conditions, which directly confirms the existence of the dynamic strengthening effect.

### 3.4. Length–Diameter Ratio of the Projectile and Curvature Radius of the Warhead

**Figure 20:**
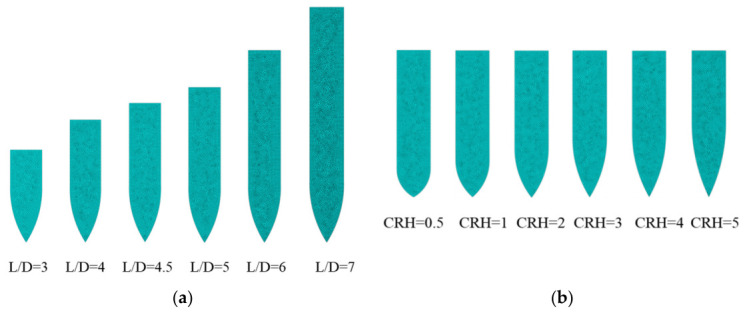
Diagram of the ratio of the length to the diameter of the projectile and the curvature of the warhead: (**a**) length–diameter ratio of the projectile; (**b**) curvature radius of the warhead.

The projectile diameter ratio refers to the ratio of the overall length L of the projectile to the diameter D of the projectile. This subsection investigates the effects of six groups of length–diameter ratios (with CRH = 4) of rigid projectiles and six groups of warhead curvature radii on the penetration process. CRH (curvature radius of the warhead) is defined as the ratio of the curvature radius of the warhead arc to the projectile diameter. The structural and dimensional parameters of each projectile are shown in Figure 20a, and the schematic diagram of projectiles with different warhead curvature radii is presented in Figure 20b.

**Figure 21:**
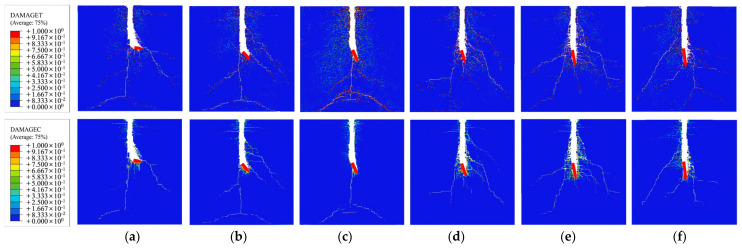
Effect of the length–diameter ratio of the projectile on the tensile and compressive damage distribution patterns of the concrete target: (**a**) L/D = 3; (**b**) L/D = 4; (**c**) L/D = 4.5; (**d**) L/D = 5; (**e**) L/D = 6; (**f**) L/D = 7.

Figure 21 illustrates the distribution of tensile damage and compressive damage in concrete targets with different projectile length–diameter ratios at t = 1500 μs. In this test series, the projectile diameter remains constant while only the projectile length is varied. A larger L/D corresponds to a higher mass and initial kinetic energy of the projectile, which results in a greater penetration depth. This regularity is consistent with the conclusion reported in the published literature [61]. Regarding projectile deflection, a larger length–diameter ratio leads to a greater deflection amplitude of the projectile. In terms of damage distribution in the target, during the penetration of a projectile with a low length–diameter ratio (L/D = 3), the target is dominated by local tensile splitting damage with a limited distribution range of cracks. In contrast, when a projectile with a high length–diameter ratio (L/D = 6) penetrates the target, the areas of tensile damage and compressive damage in the target expand significantly. Radial cracks and axial cracks interconnect with each other, and the damage mode evolves from local failure to overall damage.

**Figure 22:**
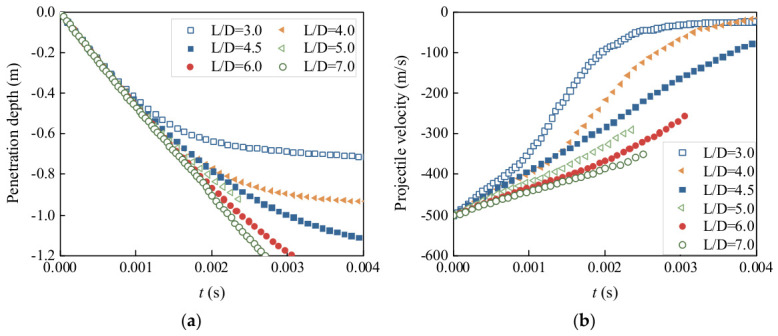
Effect of length–diameter ratio of the projectile on the penetration depth and velocity attenuation law: (**a**) penetration depth; (**b**) velocity attenuation.

Figure 22 illustrates the influence of the length–diameter ratio (L/D) on the penetration depth and the attenuation law of projectile velocity. The short and thick projectile with an L/D = 3.0 decelerates rapidly at the initial penetration stage, after which the penetration depth tends to stabilize, with a final penetration depth of only approximately 0.7 m. Under the condition of a constant projectile diameter, an increase in the length-to-diameter ratio (L/D) is accompanied by a simultaneous increase in projectile mass and initial kinetic energy, which significantly improves penetration depth and perforation capability. This conclusion is consistent with the findings reported in existing published literature [61,62,63]. The penetration depth of the projectile with an L/D = 6 is approximately 57% higher than that of the projectile with an L/D = 3, and its projectile velocity attenuation rate decreases significantly, demonstrating stronger continuous penetration capability. When L/D further increases to 6.0 and 7.0, the penetration capability of the projectile is greatly enhanced, and target penetration occurs. This phenomenon indicates that increasing the length–diameter ratio of the projectile can effectively prolong the effective penetration duration and significantly improve its penetration efficiency and ultimate penetration capability.

**Figure 23:**
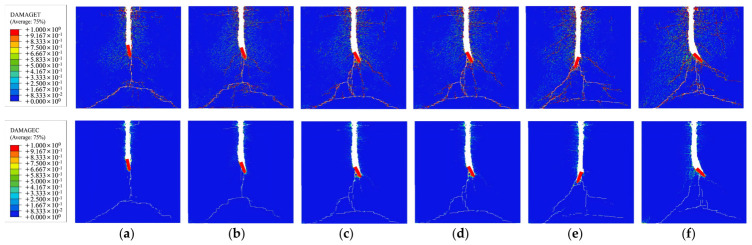
Effect of curvature radius of the warhead on the tensile and compressive damage distribution patterns of the concrete target: (**a**) CRH = 0.5; (**b**) CRH = 1; (**c**) CRH = 2; (**d**) CRH = 3; (**e**) CRH = 4; (**f**) CRH = 5.

Figure 23 presents the distribution diagrams of tensile damage and compressive damage in concrete under different CRH conditions. As CRH increases, the morphology of the tensile damage crack network evolves from being dominated by a single main crack to a multi-branched pattern. When CRH = 0.5, only one penetrating main crack and a small number of transverse secondary cracks exist in the target. When CRH increases to 3~5, a large number of oblique and transverse branch cracks emerge on the side of the projectile and in the deep layer of the target, forming a more complex crack network, with a significant increase in crack density and propagation range. This indicates that during the penetration of a sharp-nosed warhead, the affected area of tensile damage in the target is wider, and the overall degradation degree is higher. Under conditions with low CRH, the high compressive damage area is confined to the local region directly in front of the warhead. There is almost no compressive damage on the channel wall. As CRH increases, both the depth and transverse propagation range of the crushed zone increase. The high compressive damage area extends obliquely forward along the warhead shoulder, continuous compressive damage bands appear on the channel wall, and the gradient distribution of the crushed zone becomes more distinct. This indicates that the contact stress distribution of the sharp-nosed warhead is more uniform, avoiding the localization of the crushed zone caused by local stress concentration.

**Figure 24:**
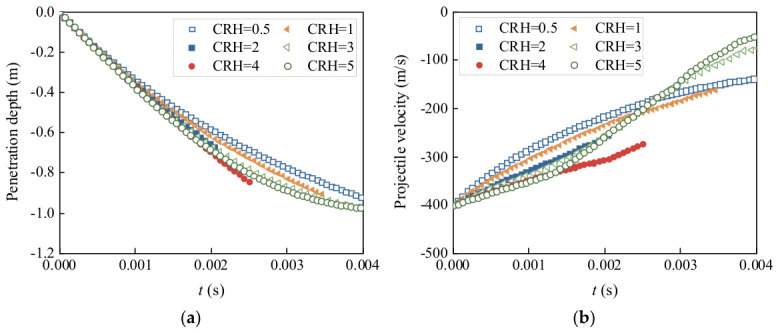
Effect of curvature radius of the warhead on the penetration depth and velocity attenuation law: (**a**) penetration depth; (**b**) velocity attenuation.

Figure 24 compares the penetration depth and velocity attenuation laws during penetration for projectiles with different CRH values. Under the same initial velocity condition, the penetration depth increases monotonically with the increase in CRH. The ogival-nose projectile with a CRH = 5 achieves the maximum final penetration depth, while the blunt-nose projectile with a CRH = 0.5 has the minimum penetration depth. As CRH increases, the time required for the projectile to reach the same penetration depth decreases, indicating that a sharper projectile nose corresponds to a shorter penetration duration and higher penetration efficiency. Figure 23b shows that the projectile velocity exhibits approximately linear attenuation over time under all conditions, but the attenuation rates differ significantly. A larger CRH corresponds to slower velocity attenuation of the projectile. The projectile with a CRH = 5 has the minimum velocity loss within the same penetration time, while the blunt nose projectile with a CRH = 0.5 exhibits the fastest velocity attenuation. It demonstrates that a sharper projectile nose can more effectively reduce the resistance during penetration and maintain a higher penetration velocity. On the whole, a sharper projectile nose (larger CRH) leads to lower penetration resistance, slower velocity attenuation, and greater penetration depth. The regulatory effect of CRH on penetration performance is particularly pronounced in the high value range (CRH = 3~5). The increase in CRH modifies the contact stress distribution between the projectile nose and the target, suppressing the stress concentration effect in the near region of the target. Meanwhile, it prolongs the effective penetration time of the projectile, ultimately resulting in an increase in penetration depth and a more complex damage pattern of the target.

## 4. Conclusions

In this study, a meso-dynamic computational framework incorporating random convex polygon aggregates and a three-phase structure is constructed by introducing a modified CDP model that accounts for the strain-rate effect. The influence mechanism of the mesoscopic parameters of the target and the geometric/motion parameters of the projectile on the penetration behavior is systematically revealed. The main conclusions are as follows:

1. Aggregate shape exerts a significant regulatory effect on penetration behavior and damage evolution. The local stress concentration induced by sharp polygonal aggregates substantially enhances crack tortuosity and the crack branching effect. It strengthens the kinetic energy dissipation mechanism by expanding the damage evolution region, thereby reducing penetration depth and accelerating velocity attenuation. For circular aggregates, the surrounding damage path tends to be straight, the damage domain is highly concentrated, and the penetration resistance of the target is relatively low.
2. Aggregate volume fraction is a key factor determining the resistance of concrete targets. As the aggregate content increases from 35% to 45%, the projectile’s penetration depth decreases, and its velocity decelerates more rapidly within the target. The mesoscale skeleton constraint within the target is enhanced, which effectively suppresses the propagation of secondary cracks and the macroscopic coalescence of main cracks. A high aggregate proportion significantly contracts the scope of tensile and compressive damage and improves the penetration resistance of concrete.
3. The initial velocity of the projectile and its geometric characteristics collaboratively dominate the damage evolution process and penetration efficiency of concrete targets. Research findings indicate that with the increase in initial velocity, the damage mode of the target evolves from local contact failure to a wide-area coupled failure. In terms of geometric parameters, an increase in the aspect ratio of the projectile significantly improves the penetration depth. An increase in the curvature radius of the projectile nose effectively reduces the opening resistance of the target, slows down velocity attenuation, and enhances the instantaneous penetration efficiency of the projectile.
4. Concrete exhibits obvious heterogeneous damage characteristics during penetration. The failure mode manifests as a coupled response of compression densification in the near field and tensile cracking in the mid-to-far field. In the middle and late stages of penetration, affected by the asymmetric resistance moment induced by the random heterogeneity of meso-structure, the projectile is prone to axial deflection, which eventually leads to the instability of the penetration trajectory and the formation of asymmetric damage zones.

This study identifies limitations in the adopted mesoscale model. The two-dimensional model neglects the real spatial geometry of aggregate particles, which may lead to underestimation of the actual penetration resistance of the target. The element deletion method adopted in this study, based on a damage threshold, can induce local mass loss and deviation from energy conservation, which may result in a certain degree of underestimation of penetration resistance. The 2 mm computational thickness of the interfacial transition zone (ITZ) adopted in this study is much larger than the actual physical thickness of the ITZ, and this setting may increase the estimated values of crack network connectivity and damage range to some extent. This effect is further amplified by the phenomenon that adjacent aggregates share the ITZ region. There are certain deviations in the applicability of the dynamic increase factor (DIF) adopted in this study when applied to high-strength and ultra-high-strength concrete.

The lack of dynamic validation is indeed one of the limitations of this research. In subsequent research prospects, further dynamic validation will be carried out based on the public penetration experiments of classic studies such as Forrestal et al. (1994) [61]. This study does not perform multiple sets of repeated calculations, and subsequent research will conduct multiple sets of stochastic simulations for typical working conditions to quantify statistical variability. Future research can introduce temperature-dependent constitutive degradation laws for each phase of the material [64], based on the mesoscale framework established in this study, to evaluate the residual penetration resistance of concrete targets after fire damage. The transition threshold of projectile trajectory between aggregate fracture and aggregate deflection can be predicted. For oblique impact and projectile deflection conditions, the existing mesoscale modeling framework does not require reconstruction of the three-phase aggregate generation algorithm, and only needs to modify the projectile incident angle. However, it is highly likely that this modification will further amplify the projectile deflection amplitude and target surface spalling range observed in vertical penetration experiments. When applied to semi-fluid domains, it is necessary to introduce an equation of state to describe the material state change under extreme high pressure, and rely on coupled Eulerian–Lagrangian methods or meshfree particle methods to overcome extreme mesh distortion.

## Figures and Tables

**Table 1:** Parameters of microscopic component materials.

Material	Young’s Modulus (GPa)	Poisson’s Ratio	Compressive Strength (MPa)	Shear Strength (MPa)
Aggregate	70	0.23	100	10
Mortar	25	0.20	35	3.5
ITZ	20	0.20	20	3.0

## Data Availability

The original contributions presented in the study are included in the article; further inquiries can be directed to the corresponding author.

## DURC Statement

The authors acknowledge that research on concrete damage and failure under penetration or extreme impact loading may have potential dual-use relevance. However, the purpose of this study is to improve the scientific understanding of mesoscopic failure mechanisms in heterogeneous concrete and to support the design, assessment, and protection of safer civil infrastructure and impact-resistant structures. This work is based on numerical simulation and does not provide operational guidance, weapon design information, or actionable recommendations for harmful use.
